# The Prognostic Significance of Tumoral Melanosis

**DOI:** 10.1111/cup.14727

**Published:** 2024-10-02

**Authors:** Alison J. Potter, Peter M. Ferguson, Serigne N. Lo, Tasnia Ahmed, Robert V. Rawson, John F. Thompson, Georgina V. Long, Richard A. Scolyer

**Affiliations:** ^1^ Melanoma Institute Australia, The University of Sydney Sydney New South Wales Australia; ^2^ Charles Perkins Centre, The University of Sydney Sydney New South Wales Australia; ^3^ NSW Health Pathology Sydney New South Wales Australia; ^4^ Faculty of Medicine and Health University of New South Wales Sydney New South Wales Australia; ^5^ Royal Prince Alfred Hospital Sydney New South Wales Australia; ^6^ Faculty of Medicine and Health The University of Sydney Sydney New South Wales Australia; ^7^ Mater Hospital & Royal North Shore Hospital Sydney New South Wales Australia

**Keywords:** immunotherapy, melanoma, melanophages, melanosis, pathology, pigmentation, prognosis, regression, treatment, tumoral

## Abstract

**Background:**

Tumoral melanosis (TM) is a histological term to describe a nodular aggregation of macrophages containing melanin pigment (melanophages) that is devoid of viable melanocytes. It is most often identified in skin, where it may be appreciated clinically as a pigmented lesion; however, it can also be found in other organs such as lymph nodes. The presence of TM is usually thought to signify the presence of a regressed melanoma or other pigmented tumor. Until recently, it was a relatively uncommon finding; however, with the use of effective systemic therapies against melanoma, its occurrence in histological specimens is more frequent.

**Methods:**

We identified and reviewed all histopathological diagnoses of TM at any organ site reported at a single institution from 2006 to 2018. TM cases were paired with non‐TM cases of cutaneous melanoma through propensity score matching at a 1:2 ratio, and their survival outcomes were compared. The clinical outcomes examined included recurrence‐free survival (RFS), distant disease‐free survival (DDFS), melanoma‐specific survival (MSS), and overall survival (OS).

**Results:**

TM was reported in 79 patients. Their median age was 65 years (range 22–88), with a 2:1 male predominance (51 out of 79, 65%). The most common organ involved was the skin (67%), with a third of all cases localized to a lower limb (36%). TM had a strong association with the presence of melanoma (91%) and regression at other sites of melanoma (54%), suggesting that it is part of a systemic immune response against melanoma. Most patients with TM either previously or subsequently developed histologically confirmed melanoma in the same anatomical region as the TM (89%). Thirty‐five TM patients were matched with 70 non‐TM cases. Patients with melanoma who developed TM without prior regional or systemic therapy showed improved MSS (*p* = 0.03), whereas no statistically significant differences were observed in terms of RFS, DDFS, and OS.

**Conclusions:**

TM usually occurs in the context of a previous or subsequent cutaneous melanoma and is associated with improved MSS. It is important that TM is recognized by pathologists and documented in pathology reports.

## 1 Introduction

**FIGURE 1:**
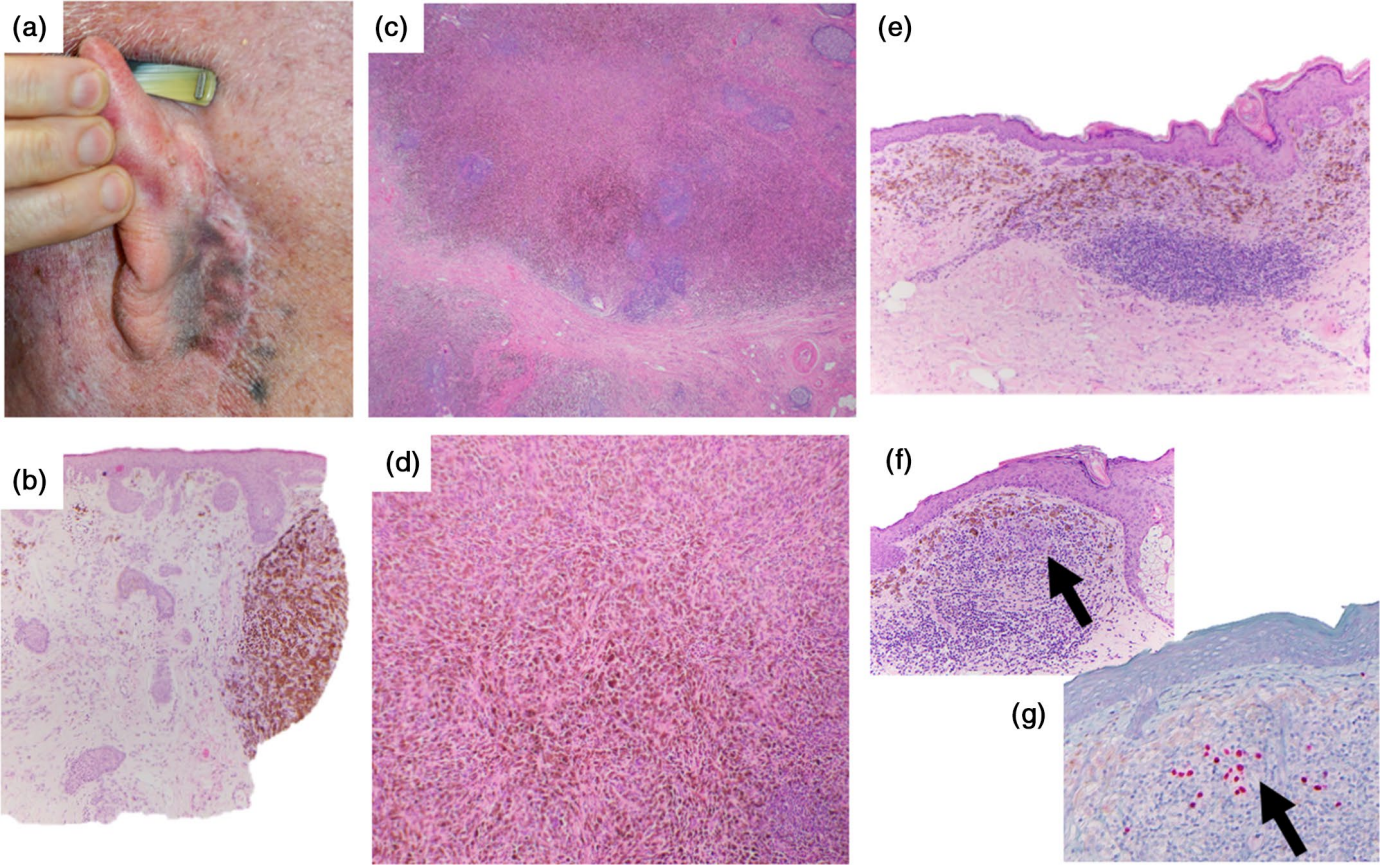
Clinical appearance of tumoral melanosis at the scar of a previous melanoma resection (a). Nodule of tumoral melanosis in the dermis (b). Tumoral melanosis in a lymph node following immunotherapy for metastatic melanoma, showing sheets of melanophages with no melanoma cells in this region (c and d). Dermal melanophages with viable melanoma cells (arrowhead), seen on H&E (e and f) and SOX‐10 immunohistochemistry (g), suggestive of partial regression.

Tumoral melanosis (TM) is a histological term that describes the presence of a nodular or sheetlike aggregation of macrophages containing melanin pigment (melanophages) that is devoid of accompanying melanocytes (Figure 1). Clinically presenting as a suspicious pigmented lesion, TM is often seen within the dermis and/or subcutis, with rare reports of lymph node deposits [1, 2, 3, 4]. TM is distinct from the diffuse pigmentation of mucosal membranes, such as in melanosis coli (characterized by deposition of lipofuscin in the intestine) [5], neurocutaneous melanosis (melanocytosis or lentigines) [6], and vulval melanosis (characterized by increased basal pigmentation sometimes accompanied by a mild increase in junctional melanocytes) [7]. TM is occasionally attributed to pigmented keratinocytic neoplasms, where melanophages are seen surrounding, or adjacent to, pigmented neoplastic epithelium such as basal cell carcinoma or squamous cell carcinoma [8]. More commonly, the nodular deposits of melanophages seen in dermal and subcuticular TM have a strong association with primary and/or metastatic melanoma [9, 10, 11].

**FIGURE 2:**
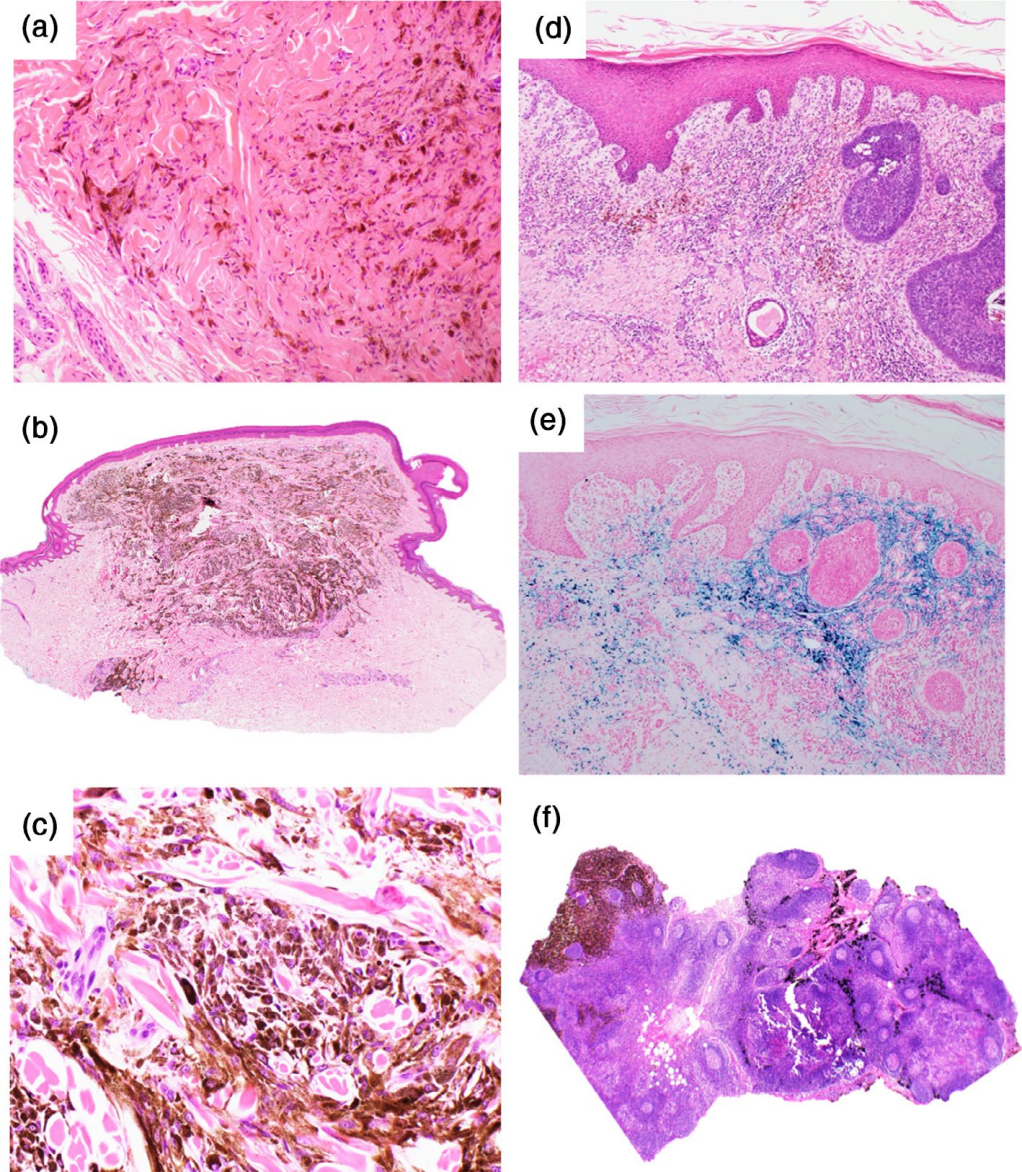
The histological differential diagnosis of tumoral melanosis (TM) includes heavily pigmented melanocytic neoplasms such as blue nevus (a) and pigmented epithelioid melanocytoma (b and c). Venous stasis dermatitis can mimic TM (d, with adjacent basal cell carcinoma), but the iron from the hemosiderin pigment is readily distinguished by the use of Perl stain (e). Tattoo pigment is comprised of black carbon particles (f) seen here in a lymph node (right) as opposed to the dark brown melanin pigment present in TM (left).

Tumoral melanosis is a diagnosis of exclusion. Other processes that lead to numerous pigment laden macrophages must be excluded, including benign melanocytic neoplasms and lesions with deposition of pigment other than melanin (see Table 1). Blue nevi (BN) are typically dermal tumors comprised of pigmented melanocytes (Figure 2a) with varying degrees of dermal fibrosis. Melanophages frequently accompany BN, particularly cellular BN, but do not tend to form confluent nodular aggregates, as seen in TM. Pigmented epithelioid melanocytomas (PEM) contain wedge‐shaped or sometimes nodular aggregates of heavily pigmented melanocytes where the presence of large epithelioid melanocytes assists in the distinction from TM (Figure 2b,c). Deep penetrating nevi (DPN, also known as WNT‐activated deep penetrating melanocytoma) are nested to plexiform proliferation of large spindled and/or epithelioid melanocytes with intercalated melanophages. In difficult cases, the application of special stains and immunohistochemistry can be helpful in BN, PEM, and DPN to identify melanocytes and classify the neoplasm. Venous stasis can present with macroscopically pigmented skin lesions with microscopic aggregations of pigmented macrophages. The dependent site, accompanying morphological changes (acanthosis/hyperkeratosis, extravasated erythrocytes, and dermal neovascularization) and the identification of hemosiderin pigment (assisted by Perl stain) will lead to the correct diagnosis (Figure 2d,e). Tattoo pigment in macrophages can collect in the skin or lymph node sinuses and may mimic TM (Figure 2f). A history of a tattoo in the draining region and black carbon pigment that stains negative for melanin (Fontana‐Masson or Schmorl) will provide a distinction from TM.

**TABLE 1:** Differential diagnosis of tumoral melanosis and ancillary tests.

Entity	Morphology	Melanin stain^a^	SOX10	Perl stain	Other IHC^b^
Tumoral melanosis	Nodular aggregates of melanophages devoid of melanocytes	+	−	−	−
Melanoma	Atypical cells with variable morphology and pigment	+	+	−	PRAME
Blue nevus	Pigmented dendritic melanocytes and melanophages	+	+	−	HMB45
Pigmented epithelioid melanocytoma	Heavily pigmented macrophages and pigment‐containing atypical epithelioid cells	+	+	−	PRKAR1A (loss)
Deep penetrating nevus	Polymorphous variably pigmented epithelioid and spindle‐shaped cells with scattered pigment‐laden macrophages	+	+	−	B‐Catenin, LEF1
Venous stasis	Hemosiderin pigment, extravasated red blood cells, dilated capillaries	−	−	+	−
Tattoo pigment	Black carbon pigment in macrophages	−	−	−	−

^a^ Schmorl or Fontana‐Masson.

^b^ Immunohistochemical stains other than SOX10 can aid diagnosis.

TM can be formed within the spectrum of histological changes attributed to regression in primary melanoma, with melanophages often seen in conjunction with a lymphocytic infiltrate and stromal fibrosis [12, 13]. The presence of regressive changes within a primary melanoma has been shown to confer a lower risk of sentinel lymph node metastasis and improved prognosis [14]. The incidence of TM has also increased since the introduction of effective systemic therapies (targeted therapies and immune checkpoint inhibitors) for metastatic melanoma, such that the qualitative evaluation of the response to neoadjuvant treatment now incorporates TM as a criterion for assessment of the response to treatment in the tumor bed [15, 16, 17].

Given the infrequent yet diverse range of clinical presentations of TM, the prognostic significance of a diagnosis of TM is poorly understood, especially when identified in patients who have not received systemic therapy. We therefore sought to characterize the clinical situations in which TM arises, describe the association between melanoma and TM, and establish the prognostic significance of identifying TM in patients with and without a prior diagnosis of melanoma.

## 2 Materials and Methods

A search was performed within the archives of the Department of Tissue Pathology and Diagnostic Oncology at Royal Prince Alfred Hospital, Sydney for histopathology reports containing “melanosis” (excluding “melanosis coli”) accessioned over a 12‐year period between 2006 and 2018. The histopathology slides and reports were reviewed by a single pathologist (P.F.), and cases were excluded if they did not meet the histological criteria of nodular (or sheetlike) aggregates of melanophages devoid of melanocytes or neoplastic cells.

Additional clinical data (including previous melanoma diagnoses, treatment modalities, and patient outcomes) were obtained from the prospectively maintained Melanoma Institute Australia Research Database. A subgroup of patients (*n* = 35, see clinical group 2 in results) was identified with a diagnosis of TM and a history of concurrent or previous melanoma treated only by surgery (without systemic therapy or radiotherapy). To assess the prognostic significance of TM on clinical outcomes, these patients were matched for sex, age, and substage of melanoma classified by the American Joint Committee on Cancer 8th Edition (AJCC 8th) [18] with a control cohort of patients who did not show histological features of TM.

Kaplan–Meier methods were applied to the matched cases and non‐TM control cohorts to mitigate potential patient selection bias. Survival differences between the matched cohorts were evaluated using the log‐rank test. Each TM case was matched with two non‐TM as control. The clinical outcomes investigated included recurrence‐free survival (RFS), distant disease‐free survival (DDFS), melanoma‐specific survival (MSS), and overall survival (OS). All statistical analysis was performed using R version 4.2.3 (R Core Team, Vienna, Austria).

The study was approved by the New South Wales Department of Health Human Research Ethics Committee (Protocol no. X15‐0454).

## 3 Results

Seventy‐nine patients with TM treated during the study period were identified. Their clinicopathological characteristics are summarized in Table 2. TM occurred in patients at a median age of 66 years (range 22–88 years) and 65% (51 out of 79) were male. Dermal TM deposits comprised half (51%, 40 out of 79) of all presentations, with a dominance in lower limbs (35%, 28 out of 79) and were infrequently seen within viscera (8%, 6 out of 79). All but seven patients (72 out of 79, 91%) had an associated history of cutaneous melanoma at the time of diagnosis of TM.

**TABLE 2:** Clinicopathological characteristics of patients with tumoral melanosis.

Characteristics	*n* = 79
Sex	
Male	51
Female	28
Age at primary diagnosis	
Mean (SD)	64 (13)
Median (range)	66 (22–88)
Anatomical site of tumoral melanosis	*n* = 79
Lower limb	28
Upper limb	15
Trunk	14
Head and neck	15
Viscera	7
Tissue location of tumoral melanosis	*n* = 79^a^
Dermis	40
Subcutis	13
Lymph node	23
Viscera	7
History of melanoma	*n* = 79
Yes	72
Melanoma treated by surgery alone	35
Melanoma treated by additional therapies other than surgery	37
No	7
Melanoma diagnosed after tumoral melanosis	2
No subsequent diagnosis of melanoma	5
Stage of melanoma—AJCC 8th edition	*n* = 62
0	1
1	10
2	11
3	37
4	13

^a^ Includes patients with multiple tissue locations of involvement.

Based on the history and subsequent management of prior invasive melanoma, patients were divided into three clinically distinct groups: (1) no history of invasive melanoma (*n* = 7), (2) a history of prior or concurrent melanoma treated by surgery alone (*n* = 35), and (3) a history of prior melanoma treated with local, regional, or systemic therapy (*n* = 37) other than surgery.

### 3.1 TM With No History of Invasive Melanoma

Seven patients presented with TM without a prior history of invasive melanoma (7 out of 79, 9%), and follow‐up information was available for 5 of them (Table 3). All cases presented with dermal TM. Two patients (Cases 4 and 5) died within 1 year of diagnosis, without reported evidence of melanoma. One patient with TM adjacent to a compound nevus on the trunk (Case 7) had no evidence of concurrent or subsequent melanoma and was alive at 6 years of follow‐up. The remaining two cases were diagnosed with melanoma within the wide local excision scar (Case 1) and/or draining lymph nodes (Case 2). Both patients were alive with no sign of recurrence (NSR) at 8 years of follow‐up.

**TABLE 3:** Characteristics of patients with tumoral melanosis and no known history of prior melanoma.

Case	Age	Sex	Site	Anatomical compartment	Follow‐up	Outcome
1	52	F	Lower limb	Dermis	Adjacent regressing melanoma in subsequent wide excision	Alive NSR 8 years
2	49	F	Lower limb	Dermis	Melanoma in draining lymph nodes	Alive NSR 8 years
3	65	M	Upper limb	Dermis	No follow‐up available	Unknown
4	69	M	Head and neck	Dermis	No evidence of melanoma	Deceased 1 year
5	78	M	Head and neck	Dermis	No evidence of melanoma	Deceased 1 year (sepsis)
6	22	F	Trunk	Dermis	No follow‐up available	Unknown
7	46	F	Trunk	Dermis	No evidence of melanoma	Alive NSR 6 years

Abbreviations: F, female; M, male; NSR, no sign of recurrent disease.

### 3.2 TM With a History of Melanoma Treated With Surgery Alone

**FIGURE 3:**
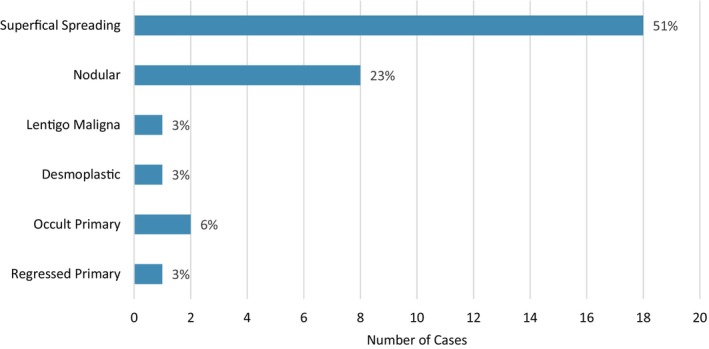
Frequency of primary melanoma types occurring in melanoma patients with TM (*n* = 35) treated with surgery alone.

Thirty‐five patients (35 out of 79, 44%) with TM had a history of melanoma treated with surgery alone (Table 4). The previous or concurrent melanoma was most frequently of superficial spreading type (51%) (Figure 3) and most patients had Stage III/IV disease at the time of TM diagnosis (60%, 21 out of 35). Melanoma was present adjacent to TM in 60% (21 out of 35), and within the same anatomical region as a melanoma in a further 29% (10 out of 35). The associated melanoma demonstrated features of regression in 54% of cases (19 out of 35), with 12 cases demonstrating features of TM at other sites (34%, 12 out of 35), including a primary melanoma (6%, 2 out of 35) and regional disease (29%, 10 out of 35). Almost half of patients developed subsequent melanoma recurrence following a diagnosis of TM (46%, 16 out of 35). Three patients were diagnosed with TM over 5 years after an initial diagnosis of melanoma, without evidence of melanoma recurrence during follow‐up.

**TABLE 4:** Characteristics of patients with tumoral melanosis and melanoma treated by surgery alone. The cases (tumoral melanosis) have been matched with controls (no tumoral melanosis) by the sex, age, and stage of their melanoma at the time of diagnosis of tumoral melanosis.

Characteristic	Controls	Cases	*p*
	(*n* = 70)	(*n* = 35)	
Sex (*n*, %)			
Female	25 (36)	11 (31)	0.83
Male	45 (64)	24 (69)	
Age at primary diagnosis (years)			
Mean (SD)	65 (15)	66 (11)	0.76
Median (range)	67 (21–97)	67 (40–85)	
Stage (AJCC 8) (*n*, %)			
1	15 (21)	7 (20)	0.74
1A	9	3	
1B	6	4	
2	13 (19)	7 (20)	
2A	3	3	
2B	4	1	
2C	6	3	
3	24 (34)	15 (43)	
3A	3	1	
3B	5	5	
3C	13	9	
3D	3	0	
4	18 (26)	6 (17)	
Treatment following (*n*, %)	28^a^ (40)	14^a^ (40)	
Immunotherapy	17 (24)	8 (23)	0.18
BRAF‐targeted therapy	10 (14)	3 (9)	
Radiotherapy	19 (27)	6 (17)	

^a^ Some patients received multiple treatment modalities.

**FIGURE 4:**
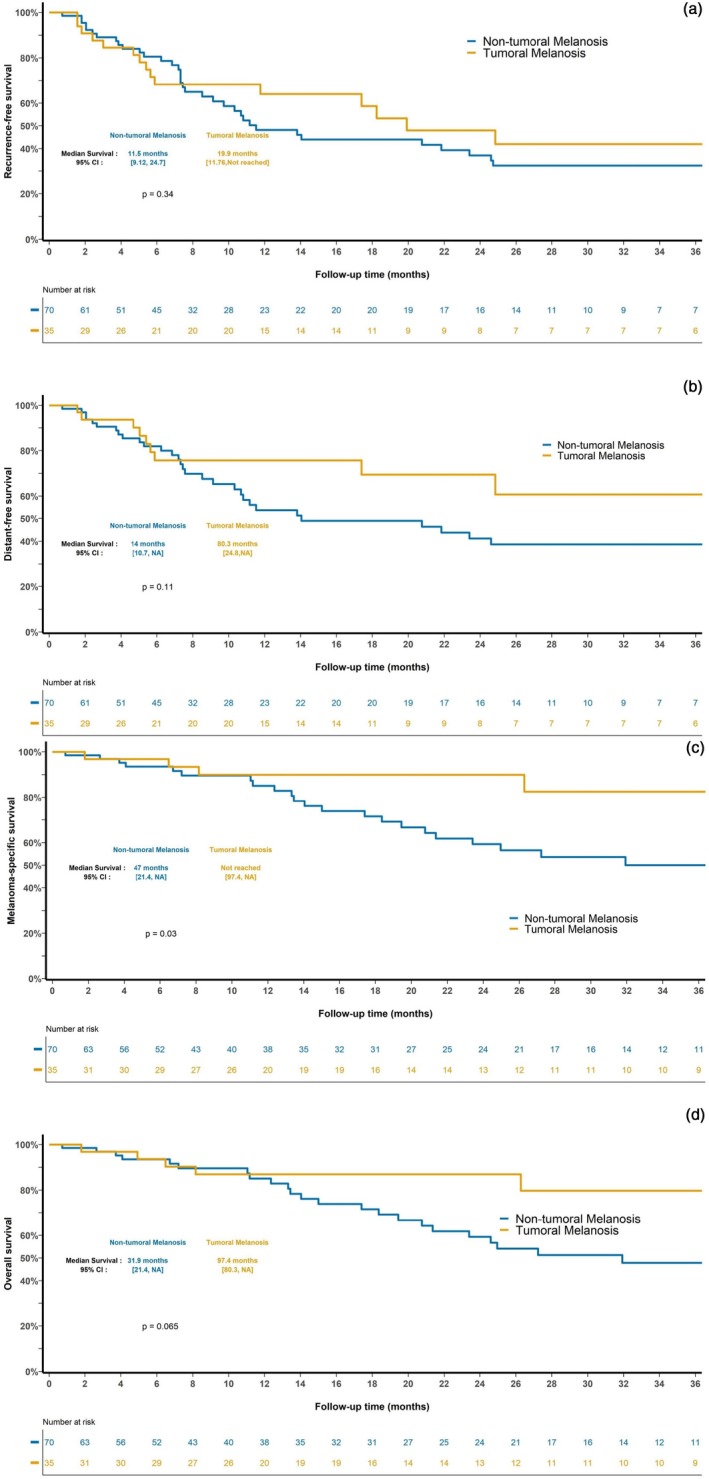
Kaplan–Meier survival estimates for melanoma patients with TM (*n* = 35) treated with surgery alone compared to matched control melanoma patients without TM (*n* = 70). (a) Recurrence‐free survival, (b) distant disease‐free survival, (c) melanoma‐specific survival, and (d) overall survival. Differences in survival were tested using the log‐rank test.

Patients with a history of melanoma treated with surgery alone (*n* = 35) showed no significant differences in sex, age, stage of melanoma, or treatment regimen from the matched control cohort (*n* = 70) (Table 4). Despite the higher relative percentage of Stage 4 patients in the control group (26% vs. 17%), the distribution of melanoma stages across the TM and non‐TM matched cohorts was not statistically significant. Using a Fisher test, we found no impact on MSS difference between cases and controls with Stage 4 disease to confirm that the higher proportion of stage 4 patients did not affect the survival outcomes. Melanoma patients diagnosed with TM had similar recurrence‐free (*p* = 0.34) and distant metastasis‐free survival rates to matched controls without TM (*p* = 0.11) (Figure 4a,b). Patients with TM had significantly improved MSS (*p* = 0.03) (Figure 4c,d). The presence of TM was associated with differences in OS (median survival 97.4 months vs. 31.9 months) that were not statistically significant (*p* = 0.065).

### 3.3 TM With a History of Melanoma Treated With Local (Non‐Surgical) or Systemic Therapy (*n* = 37)

Thirty‐seven patients (37 out of 79, 47%) with TM had a history of melanoma treated with regional or systemic therapy (targeted or immunotherapy). TM was most frequently seen in patients treated with immune checkpoint inhibitor therapy before biopsy (24 out of 37, 64%), two of whom had also received targeted therapy (combination BRAF/MEK inhibition) during their disease course. Eight patients received targeted therapy alone (22%, 8 out of 37). Regional therapies received by five patients included Rose Bengal injections and radiotherapy (1 out of 37), isolated limb infusion (cytotoxic chemotherapy, 2 out of 37), and topical immunotherapy (diphencyprone, 2 out of 37). Clinical follow‐up was not available for this heterogeneous group.

## 4 Discussion

To the best of our knowledge, this study reports the largest single‐institution cohort of patients with TM published to date [16]. Our data confirm a strong association of TM with primary and metastatic melanoma (93% of patients) within three distinct clinical subgroups, with differing prognostic implications following a TM diagnosis.

TM usually presents in patients with a history of melanoma (72 out of 79, 91%). One patient within our cohort did not develop any subsequent melanoma (Case 7) after 6 years of follow‐up, in line with similar case reports [19, 20]. The presence of TM alone is not necessarily indicative of melanoma, with a similar phenomenon described adjacent to pigmented keratinocytic or hematological neoplasms, as well as benign melanocytic nevi [8, 21, 22]. As has been reported previously, two cases within our cohort demonstrated features of cutaneous TM, with viable melanoma only identified at regional and distant sites following thorough clinical assessment and management [23]. The diagnosis of isolated TM in these cases could represent under‐sampling of the original lesion, since 60% of patients in Group 2 (with a history of surgically managed melanoma) demonstrated melanoma adjacent to TM at the time of diagnosis. Alternatively, such cases could represent complete regression of a melanoma that has already metastasized. Nevertheless, it would appear prudent for patients diagnosed with isolated TM to undergo an extensive clinical review for primary or metastatic melanoma, including thorough cutaneous and mucosal examination, assessment of regional node fields, and histological examination of any additional suspicious lesions identified. In addition, partially sampled TM should be followed by complete surgical excision of the affected site, as viable melanoma can be present in the adjacent tissue (as seen in Case 1, Table 3).

TM can occur as a component of a stepwise inflammatory host response targeted towards melanoma, termed “regression.” Rarely, it may occur in the latter stages of regression, where it is preceded by a tumor‐associated lymphocytic infiltrate accompanied by an immature fibroblastic stromal reaction. The host reaction culminates as epidermal atrophy, dense dermal fibrosis and pigment incontinence, which occasionally may manifest as TM [12]. Diagnosis of TM is considered when melanophages form nodules or sheets, and Jurgen et al. propose a minimum size threshold of 5 mm [16]. However, a recent case series reported an average TM size of 4.8 mm with more than a third of cases > 3.5 mm [24]. No cases of TM with a diameter < 2.5 mm were encountered in this previous study or the present, and we recommend this as a more accurate minimum threshold for a diagnosis of TM.

The presence of regressive features within a primary lesion is a favorable prognostic feature, as shown by both systematic review and large cohort studies demonstrating improved RFS and OS in patients with T1 and T2 melanomas [25, 26]. Regression also predicts having a negative sentinel lymph node biopsy (odds ratio [OR] = 0.33, 95% confidence interval [CI] = 0.20–0.52, *p* < 0.001) when assessed in conjunction with tumor‐infiltrating lymphocytes [14]. Of the 35 patients who had their melanoma surgically managed in the current study, over half (54%, 19/35) had features of regression within their prior melanoma, and almost a third (34%, 12/35) demonstrated multiple foci of TM, suggesting a systemic immune‐mediated reaction to additional sites of disease beyond the primary melanoma. Supporting an enduring prognostic benefit of this response using a case‐controlled cohort, this is the first study to demonstrate that the diagnosis of TM deposits confers an improved MSS (*p* = 0.03). However, the presence of TM in these patients failed to influence the risk of recurrence and metastasis, and thus patients presenting with TM still warrant close clinical follow‐up for the development of recurrence at new sites of active disease.

The incidence of TM has increased in the last decade, following the introduction of effective systemic therapies for the management of metastatic melanoma. Seventeen case reports of TM have been published in the last 5 years, following the use of anti‐PD‐1 therapies (pembrolizumab and nivolumab) or anti‐CTLA‐4 therapy (ipilimumab) in patients with resected AJCC 8th Edition Stage III/IV disease [16, 27, 28, 29, 30, 31, 32, 33]. TM may also be seen in patients treated with targeted BRAF inhibitors with or without concurrent MEK inhibition [34, 35]. We similarly identified a group of patients receiving either immunotherapy or targeted therapy as well as a subset of patients who received locoregional therapies with cytotoxic or immunomodulatory effects. The presence of TM (pigment‐laden macrophages) has been well‐documented in clinical trials of neoadjuvant systemic therapy for Stage III melanoma, with 69% of patients receiving targeted therapy [36] and 57% of patients receiving immunotherapy [17] showing features of TM within the tumor bed at treated sites of disease. Identification of TM in patients receiving immunotherapy is not predictive of a favorable clinical response, with prior cases describing disease progression despite the presence of TM at sites of treated disease [16]. Neoadjuvant therapy trials in Stage III patients showed that while the presence of hyalinized fibrosis correlated with improved RFS following targeted therapy (hazard ratio = 0.4, 95% CI = 0.2–0.9, *p* = 0.014) and immunotherapy (OR = 0.89, 95% CI = 0.79–0.96, *p* = 0.025). TM was not correlated with survival differences for either form of therapy [17, 36]. The prognosis of patients demonstrating TM at sites of disease, including those identified within the current study, would be more likely dependent on additional clinical and histopathological predictors of treatment response, including volume of residual disease and possible development of hyaline‐type fibrosis within the tumor bed, which has been demonstrated to represent a more effective immune response against the tumor and to be associated with a prolongation RFS in neoadjuvant therapy trials in Stage III melanoma [17, 36]. Furthermore, our study is not designed to assess whether TM in treatment naïve patients is predictive of a response to immunotherapy.

While this is the largest series of TM reported to date as far as we are aware, the study was limited by its retrospective design and imperfect control of extraneous variables inherent in case–control studies. We recognize that the anatomical distribution of involved sites reported in our study is heavily influenced by ease of detection at cutaneous sites, clinical suspicion of active disease, sites amenable to biopsy or excision, and extent of disease detected on clinical or radiological examination. Patients monitored with positron emission tomography (PET), especially following systemic therapy, may present with multiple sites of metabolic activity attributed to active disease, which may otherwise represent unsampled visceral deposits of inflammation [37] or TM [38]. Pseudo‐progression identified radiologically often remains unsampled for histopathological diagnosis, instead relying on serial imaging or emerging biomarkers such as circulating tumor DNA to establish an absence of disease [39]. Therefore, nodal and visceral presentations of TM in both surgically‐ and systemically treated melanoma patients may be under‐represented in this study.

In conclusion, TM usually occurs in patients with known melanoma or in whom melanoma subsequently becomes manifest. It is seen in three clinical contexts, each with unique clinical and prognostic implications. Diagnosis of TM by pathologists is essential for the appropriate clinical assessment of patients without a prior history of melanoma. As a feature of tumor regression, TM is a diagnostic feature of spontaneous regression with positive prognostic implications. Recognition of TM as a component of regression with the tumor bed following systemic therapy is also crucial for accurate evaluation of therapy effect and consideration of ongoing management strategies. Furthermore, in the treatment‐naive setting, TM is predictive of improved MSS and thus provides prognostic information in addition to standard staging criteria. Therefore, in a range of clinical contexts, the recognition of TM by pathologists can provide important information about melanoma risk and prognosis.

## Disclosure

The authors have nothing to report.

## Conflicts of Interest

Serigne N. Lo has received an honorarium for editorial duties from The British Association of Dermatologists. John F. Thompson has received honoraria for advisory board participation from BMS Australia, MSD Australia, GSK, and Provectus Biopharmaceuticals, and travel and conference support from GSK, Provectus Biopharmaceuticals and Novartis. Georgina V. Long is consultant advisor for Agenus, Amgen, Array Biopharma, AstraZeneca, Bayer, BioNTech, Boehringer Ingelheim, Bristol Myers Squibb, Evaxion, Hexal AG (Sandoz Company), Highlight Therapeutics S.L., IOBiotech, Immunocore, Innovent Biologics USA, Merck Sharpe & Dohme, Novartis, PHMR Ltd., Pierre Fabre, Regeneron, Scancell, and SkylineDX B.V. Richard A. Scolyer has received fees for professional services from SkylineDx BV, IO Biotech ApS, MetaOptima Technology Inc., F. Hoffmann‐La Roche Ltd., Evaxion, Provectus Biopharmaceuticals Australia, Qbiotics, Novartis, Merck Sharp & Dohme, NeraCare, AMGEN Inc., Bristol‐Myers Squibb, Myriad Genetics, and GlaxoSmithKline. The other authors declare no conflicts of interest.

## Data Availability

The data that support the findings of this study are available on request from the corresponding author. The data are not publicly available due to privacy or ethical restrictions.
